# Epidermal glucocorticoid and mineralocorticoid receptors act cooperatively to regulate epidermal development and counteract skin inflammation

**DOI:** 10.1038/s41419-018-0673-z

**Published:** 2018-05-22

**Authors:** Judit Bigas, Lisa M. Sevilla, Elena Carceller, Julia Boix, Paloma Pérez

**Affiliations:** Instituto de Biomedicina de Valencia-Consejo Superior de Investigaciones Científicas (IBV-CSIC), Jaime Roig 11, 46010 Valencia, Spain

## Abstract

Endogenous and synthetic glucocorticoids (GCs) regulate epidermal development and combat skin inflammatory diseases. GC actions can be mediated through the GC receptor (GR) and/or the mineralocorticoid receptor (MR), highly homologous ligand-activated transcription factors. While the role of GR as a potent anti-inflammatory mediator is well known, that of MR is not as clear, nor is whether these receptors cooperate or antagonize each other in the epidermis. To address this, we generated mice with epidermal-specific loss of both receptors (double knockout, DKO), and analyzed the phenotypical and functional consequences relative to single KOs or controls (CO). At birth, DKO epidermis displayed a phenotype of defective differentiation and inflammation, which was more severe than in either single KO, featuring neutrophil-containing infiltrates, and gene dysregulation characteristic of human psoriatic lesions. This phenotype resolved spontaneously. However, in adulthood, single or combined loss of GC receptors increased susceptibility to inflammation and hyperproliferation triggered by phorbol ester which, different to CO, was not effectively counteracted by GC treatment. Also, DKOs were more susceptible to imiquimod-induced psoriasis than CO showing severe defective epidermal differentiation and microabcesses while single KOs showed an intermediate response. Immortalized DKO keratinocytes featured increased proliferation kinetics and reduced cell size, a unique phenotype relative to single KO cells. The lack of GR and MR in keratinocytes, individual or combined, caused constitutive increases in p38 and ERK activities, which were partially reversed upon reinsertion of receptors into DKO cells. DKO keratinocytes also displayed significant increases in AP-1 and NF-κB transcriptional activities, which were partially rescued by ERK and p38 inhibition, respectively. Reinsertion of GR and MR in DKO keratinocytes resulted in physical and cooperative functional interactions that restored the transcriptional response to GCs. In conclusion, our data have revealed that epidermal GR and MR act cooperatively to regulate epidermal development and counteract skin inflammation.

## Introduction

Glucocorticoid (GC) derivatives are the most effective and widely prescribed compounds for treating inflammatory and autoimmune diseases. However, their therapeutic use is limited by the adverse side-effect profile that in skin includes epidermal thinning, dermal atrophy, impaired wound healing and increased fragility, dehydration and infection risk^1–3^. These adverse effects are similar to symptoms of individuals with abnormally high endogenous production of GCs (Cushing’s syndrome) as well as in the elderly population. GC deficiency (Addison’s disease, also featuring mineralocorticoid deficiency) also results in skin alterations^4,5^, highlighting the requirement for appropriate GC levels for normal tissue function.

The skin prevents dehydration, mechanical trauma, and infection^6^. The epidermis, the epithelial compartment of the skin, is mainly composed of keratinocytes which undergo terminal differentiation to generate the dead, flattened squames of the stratum corneum (SC), required for barrier function^6,7^. Defects in differentiation are associated with inflammation as a faulty epidermal barrier allows the entrance of allergens that stimulate the immune response leading to the prevalent inflammatory skin disorders atopic dermatitis and psoriasis^8,9^.

GC synthesis in the adrenal cortex is controlled by the hypothalamic–pituitary–adrenal (HPA) axis^10^. GCs exert their effects through binding to the GC receptor (GR) and the mineralocorticoid receptor (MR), structurally and functionally homologous ligand-activated transcription factors^1,11–13^. In response to endogenous hormones and synthetic ligands, GR and MR dissociate from multimeric cytoplasmic inhibitory complexes, undergo post-translational modifications, translocate to the nucleus and bind to GC response elements (GREs) in target genes.

The therapeutic actions of GC-activated GR occur through distinct mechanisms, including: (i) physical interaction (tethering) with pro-inflammatory transcription factors such as NF-κB and AP-1, independent of DNA-binding; (ii) antagonism with MAPKs p38, ERK, and JNK, which act upstream of AP-1 and in the case of p38, NF-κB; and (iii) transcriptional induction of anti-inflammatory genes (e.g., *Dusp1/Mkp1* and *Tsc22d3/Gilz*) by binding to GREs^1,11,14^.

GR is ubiquitously expressed, however, MR shows a more restricted expression pattern with highest levels in kidney and cardiovascular tissues^11–13^. Both receptors are expressed in the epidermis and its appendages^5,15–18^. Importantly, MR has a 10-fold higher affinity for GCs than GR. Expression of the GC-inactivating enzyme 11-beta hydroxysteroid dehydrogenase (HSD11B) type 2 in tissues such as kidney and heart protects MR from constant GC occupation and allows activation by aldosterone, which has key roles in electrolyte balance^10,13^. In these tissues, MR activation is involved in pathophysiological effects ultimately leading to inflammation and fibrosis and MR antagonists are beneficial for patients with cardiovascular and renal disease^19^. However, it is currently accepted that MR exerts a broader range of functions in non-classical tissues, such as in the brain, and that its activation can be beneficial^19^.

Our previous studies addressed the consequences of epidermal loss of either GR (GR epidermal KO or GR^EKO^)^15^ or MR (MR epidermal KO or MR^EKO^)^16^ in skin. Newborn GR^EKO^ mice showed an impaired epidermal barrier with increased proliferation and defective differentiation while MR^EKO^ mice showed minor defects. Numerous genes were upregulated in GR^EKO^ epidermis which are overexpressed in psoriatic patients, however, these alterations resolved spontaneously early after birth^15^ suggesting MR may have a compensatory role.

Adult GR^EKO^ and MR^EKO^ mice featured minor skin defects but had worsened responses to inflammatory triggers compared to controls (CO) demonstrating that both epidermal GR and MR act as anti-inflammatory mediators^15,16^. However, whether GR and MR cooperate to counteract skin inflammation was unknown.

In this study, the generation and characterization of mice and cell lines lacking epidermal expression of both GR and MR (GR^EKO^/MR^EKO^ double knockout, DKO) has revealed cooperation between these receptors during development as well as additive anti-inflammatory roles in diseased skin.

## Results

### Combined loss of GR and MR severely impairs epidermal development

**Fig. 1:**
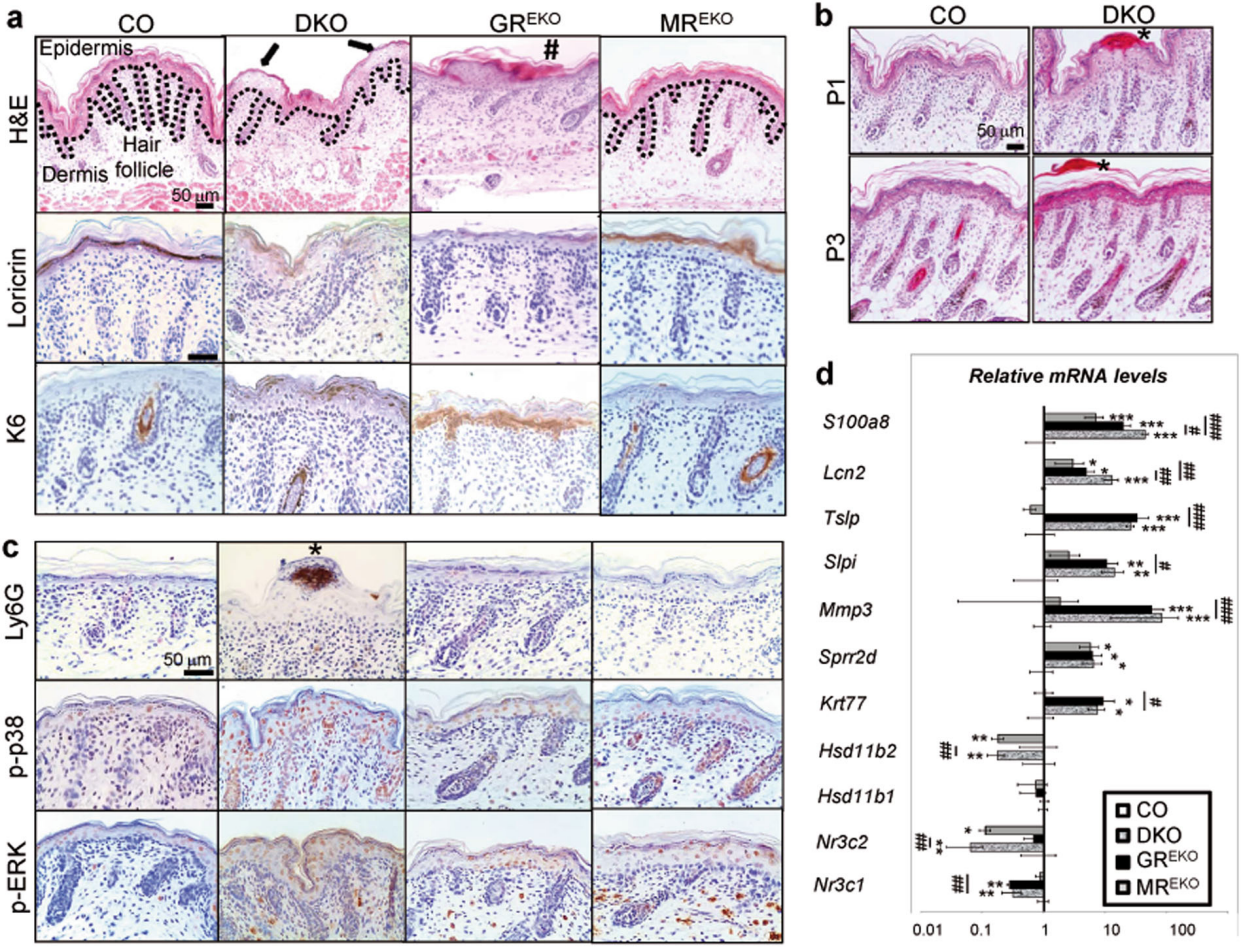
Severe impaired epidermal differentiation and inflammation, increased phosphorylated-MAPKs, and gene dysregulation upon combined loss of GR and MR. **a** Hematoxylin & eosin (H&E)-stainings (top) and loricrin and keratin(K)6 immunohistochemistry (middle, bottom) of skin sections from postnatal day (P) 0. Control (CO), GR epidermal knockout (GR^EKO^), MR epidermal knockout (MR^EKO^) or combined GR and MR epidermal knockout (DKO). Dotted line separates epidermis and dermis. Arrows: defective differentiation and minimal stratum corneum; hash: altered stratum corneum. **b** H&E-stained sections of CO and DKO mouse skin at the indicated postnatal days (P1, P3). Asterisks: microabscesses. **c** Immunostaining for Ly6G, phospho(p)-p38, and p-ERK of mouse skin from CO, DKO, GR^EKO^, and MR^EKO^ P1 littermates. Asterisks: neutrophil-containing microabscesses. **d** Gene expression was quantitated by RT-QPCR using epidermal RNA from P0 DKO, GR^EKO^, and MR^EKO^ mice (relative to CO). Asterisks indicate significant differences relative to CO and hashes significant differences between DKO and single KO: *^, #^ < 0.05, ** < 0.01, ***^, ###^ < 0.001; *n* > 3 in each experimental group

To understand the relative roles of GR and MR in skin homeostasis, we generated DKO mice with epidermal-specific loss of both receptors, and analyzed the phenotypical and functional differences relative to single KO or CO mice (Fig. 1).

DKOs were viable and fertile. At postnatal day 0 (P0), DKOs displayed a striking phenotype of defective epidermal differentiation, more severe than in GR or MR single KOs, with patches of decreased granular layer and minimal SC (Fig. 1a, arrows). Morphological changes were evident, with keratinocytes appearing disorganized and failing to flatten suprabasally. The differentiation marker loricrin showed reduced patchy expression in both GR^EKO^ and DKO compared to MR^EKO^ and CO (Fig. 1a). Both DKO and GR^EKO^ showed aberrant epidermal expression of Keratin (K)6, though in the basal layer in GR^EKO^ and the granular layer of DKO (Fig. 1a).

The epidermal alterations in P0 DKOs were likely due to a delay in differentiation since SC was apparent at P1 (Fig. 1b). Remarkably, we detected epidermal microabscesses in DKOs, a phenotype not observed in single KO or CO mice (Fig. 1b, c; asterisks). Microabscesses are associated with inflammatory pathologies such as psoriasis and usually contain neutrophil infiltrates^20^. Indeed, we detected Ly6G-positive cells in DKO microabscesses (Fig. 1c). By P3, the epidermis of DKO animals appeared almost entirely normal, and microabscesses were being lost through desquamation (Fig. 1b). We also detected increased levels of the phosphorylated (p-) forms of p38 and ERK in the epidermis of P0 DKO and single KOs relative to CO (Fig. 1c).

We assessed the expression of genes involved in epidermal development (*Krt77*, *Sprr2d*) and inflammation (*Mmp3, Slpi, Tslp*, *Lcn2*, and *S100a8*) and found increased mRNA levels in DKO vs. CO newborn epidermis (Fig. 1d). Similar dysregulation of *Krt77*, *Sprr2d*, *Mmp3, Slpi*, and *Tslp* was observed in GR^EKO^ but not MR^EKO^ epidermis, suggesting GR-dependent regulation (Fig. 1d). On the other hand, *Hsd11b2* was drastically reduced in both DKO and MR^EKO^—but not GR^EKO^—suggesting dependence on MR (Fig. 1d). *Sprr2d* was increased in all KOs suggesting involvement of both receptors in its regulation while *Hsd11b1* expression was not affected by the loss of either or both receptors (Fig. 1d). Importantly, *Lcn2* and *S100a8* were upregulated in all KOs with additive increases in DKO relative to single KOs, suggesting cooperative actions (Fig. 1d).

The skin phenotype of DKOs resolved around P5 and adult skin sections showed no major differences relative to CO except for an increase in epidermal thickness (Fig. S1a). These alterations were similar to those observed in each single KO^16,17^ suggesting similar roles for GR and MR in adult skin homeostasis.

However, the transcriptional response of DKO skin to topically applied dexamethasone (Dex) was absent as there was no induction of GC-target genes *Zfp36*, *Ddit4*, and *Fkbp51* or repression of *Ccnd1* (Fig. S1b). These data indicate that the absence of GR and MR in epidermis, even though other skin cells have intact expression of both receptors, is sufficient to impair the GC-dependent transcriptional activity in skin.

### Adult DKO mice show increased PMA-induced skin inflammation

**Fig. 2:**
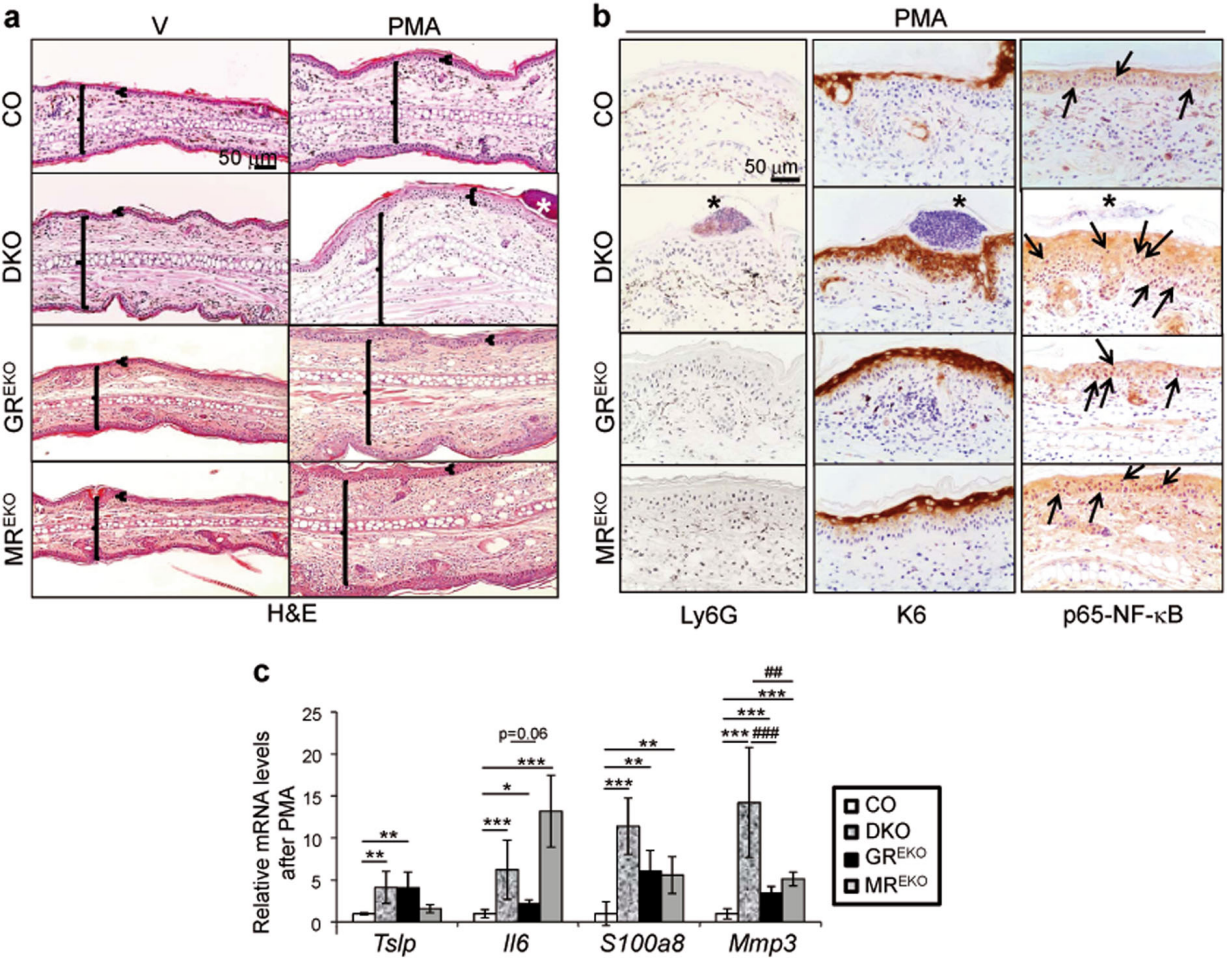
Increased PMA-induced skin inflammation in DKO mice. **a** Representative images of H&E-stained sections of CO, DKO, GR^EKO^, and MR^EKO^ mouse ears after topical treatment with the phorbol ester 12-myristate 13-acetate (PMA) or vehicle (V). Large brackets: ear edema; small brackets: epidermal thickness; asterisks: neutrophil-containing microabscesses. **b** Immunostaining for Ly6G, K6, and p65-NF-κB in skin samples from (**a**). **c** The relative mRNA levels of *Tslp*, *Il6*, *S100a8*, and *Mmp*3 in PMA-treated CO, DKO, GR^EKO^, and MR^EKO^ epidermis were quantitated by RT-QPCR. Asterisks indicate statistically significant differences relative to CO and hashes significant differences of DKO relative to single KO: * < 0.05, **^, ##^ < 0.01, ***^, ###^ < 0.001; *n* > 4 mice per genotype

Next, we assessed the consequences of combined epidermal loss of GR and MR in inflammation in vivo by inducing ear edema via topical application of the phorbol ester 12-myristate 13-acetate (PMA). PMA treatment significantly increased edema and epidermal thickness in DKOs—but not in single KOs—relative to CO (Fig. 2a, and S2). Importantly, only DKO ears displayed epithelial microabscesses containing neutrophils after PMA treatment (Fig. 2b, Ly6G, asterisks). We also detected increased interfollicular expression of K6 in PMA-treated DKO and single KOs vs. CO skin. However, while K6 was detected throughout all epidermal layers in the DKO it was restricted to suprabasal keratinocytes in the single KOs (Fig. 2b). In PMA-treated DKO and single KO skin, the nuclear p65-NF-κB was strongly increased in basal and suprabasal keratinocytes relative to CO (Fig. 2b, arrows).

Consistent with these results, PMA triggered increased upregulation of the inflammatory markers *Il6*, *S100a8*, and *Mmp3* in DKO and single KOs relative to CO ear epidermis (Fig. 2c). While *Tsl*p was induced only in GR^EKO^ and DKO, *Il6* showed the strongest upregulation in MR^EKO^ epidermis. There was an increased trend of *S100a8* expression in DKO as compared to GR^EKO^ or MR^EKO^ although it did not reach statistical significance; however *Mmp3* expression showed additive increases in DKO relative to single KOs (Fig. 2c).

We asked whether the individual or combined absence of epidermal GR and MR reduced the therapeutic effects of GCs on skin inflammation by treating dorsal skin with PMA alone or in combination with Dex. In CO mice, Dex reduced the PMA-induced epidermal thickening, K6 expression, and the number of BrdU-positive proliferating keratinocytes (Fig. S3a–c). In contrast, these effects of Dex were drastically reduced in DKOs and single KOs (Fig. S3a–c). Also, the increased nuclear p65-NF-κB in DKO and single KO epidermis remained elevated even after PMA plus Dex treatment (Fig. S3b). These results show that both epidermal GR and MR are required for the anti-proliferative and protective actions of GCs in inflamed skin.

### Increased sensitivity of DKO to IMQ-induced psoriasis correlates with decreased expression of GC targets

We next investigated the consequences of epidermal-specific GR and MR loss in psoriasis using the imiquimod (IMQ)-induced protocol which consists in topical repetitive applications of Aldara®, a cream containing 5% of the toll-like receptor 7 (TLR7)-agonist IMQ, which induces histopathological and molecular changes that closely recapitulate the human disease^21^.

**Fig. 3:**
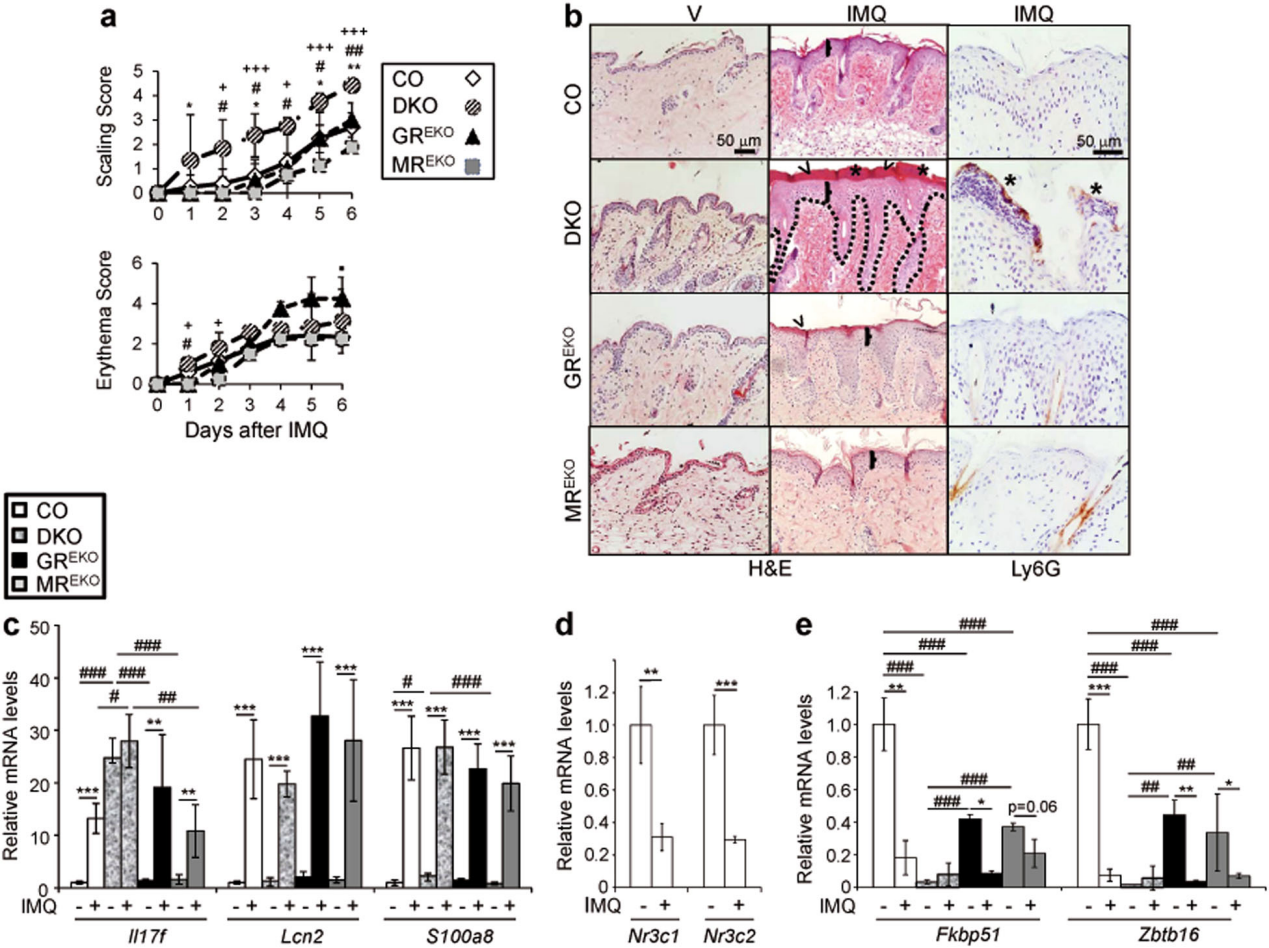
Increased sensitivity of DKO to IMQ-induced psoriasis correlates with decreased expression of GC targets. **a** Score of scaling and erythema in CO, DKO, GR^EKO^, and MR^EKO^ mice treated with imiquimod (IMQ) for 6 days. *, differences of DKO relative to CO; #, differences of DKO relative to GR^EKO^; +, differences of DKO relative to MR^EKO^; ·, differences of GR^EKO^ relative to CO; *^, #, +, ·^*p* < 0.05; **^, ##, ++^*p* < 0.01; ^+++^*p* < 0.001; *n* > 5 mice per experimental group. **b** Representative images of skin sections of vehicle (V) and IMQ-treated mice that were H&E-stained or immunostained for Ly6G. Brackets: epidermal thickening; arrowheads, abnormal keratinocyte differentiation; and asterisks, immune infiltrates containing neutrophils. **c**–**e** Relative mRNA levels for indicated genes were quantitated by RT-QPCR in V and IMQ-treated skin of the indicate genotypes. *, comparison between treatments within one genotype; #, comparison between genotypes within the same treatment; *^, #^*p* < 0.05, **^, ##^*p* < 0.01, ***^, ###^*p* < 0.001; *n* > 4 in each experimental group

Mice were treated with IMQ and erythema and scaling were scored daily, with increases in desquamation in DKO relative to CO or single KOs observed from day 3 onwards (Fig. 3a and S4). DKOs showed an increased response to IMQ relative to CO, GR^EKO^ or MR^EKO^ mice, including increased epidermal thickening and abnormal keratinocyte differentiation (retention of the nuclei in the SC or parakeratosis; Fig. 3b, brackets, and arrowheads, respectively). Focal loss of HSD11B2, normally expressed in differentiated epidermis was observed in DKO and GR^EKO^ (Fig. S5). Immunostaining of IMQ-treated skin sections showed increases in p-p38 and p-ERK in the epidermis of all KOs relative to CO animals (Fig. S5). Importantly, DKOs showed features of increased disease severity not apparent in single KOs, including abundant immune infiltrates containing neutrophils resembling Munro-like abscesses in psoriatic lesions (Fig. 3b, Ly6G).

Although the absence of GR and MR in keratinocytes did not cause spontaneous psoriasis *per se*, we detected constitutive upregulation of several genes contributing to the disease such as *Il17f*, *Il22*, and *Il23* in vehicle-treated DKO skin relative to single KO or CO (Fig. 3c, and data not shown). After IMQ treatment *Il17f* levels increased in CO and single KO and remained high in DKOs. However, the expression of the psoriasis marker *Lcn2* did not differ between genotypes in basal or disease conditions while *S100a8* was 2-fold upregulated in vehicle-treated DKO vs. CO and MR^EKO^ (Fig. 3c).

Recent findings reported decreases in mRNA levels of GR/*NR3C1* and MR/*NR3C2*, as well as GC-target genes *FKBP51* and *ZBTB16* in human psoriatic lesions^22,23^. Consistently, we detected strong downregulation of *Nr3c1* and *Nr3c2* as well as *Fkbp51* and *Zbtb16* in IMQ-treated CO skin (Fig. 3d, e). Importantly, *Fkbp51* and *Zbtb16* were constitutively downregulated in DKO relative to CO mice (>20-fold) and IMQ treatment did not further decrease their expression (Fig. 3e). In GR^EKO^ and MR^EKO^, *Fkbp51* and *Zbtb16* also showed decreased basal expression relative to CO (2.5- to 3-fold) and IMQ reduced their expression to a similar extent as in CO (Fig. 3e).

### Immortalized DKO keratinocytes show a unique phenotype relative to GR^EKO^, MR^EKO^, and CO

To unequivocally decipher the cell-type specific roles of GR and MR, we established immortalized DKO keratinocyte cell lines from adult epidermis (Fig. S6), and performed comparative assessment of their morphology, degree of differentiation, and growth kinetics relative to previously established single KO and CO cells^16,24^.

**Fig. 4:**
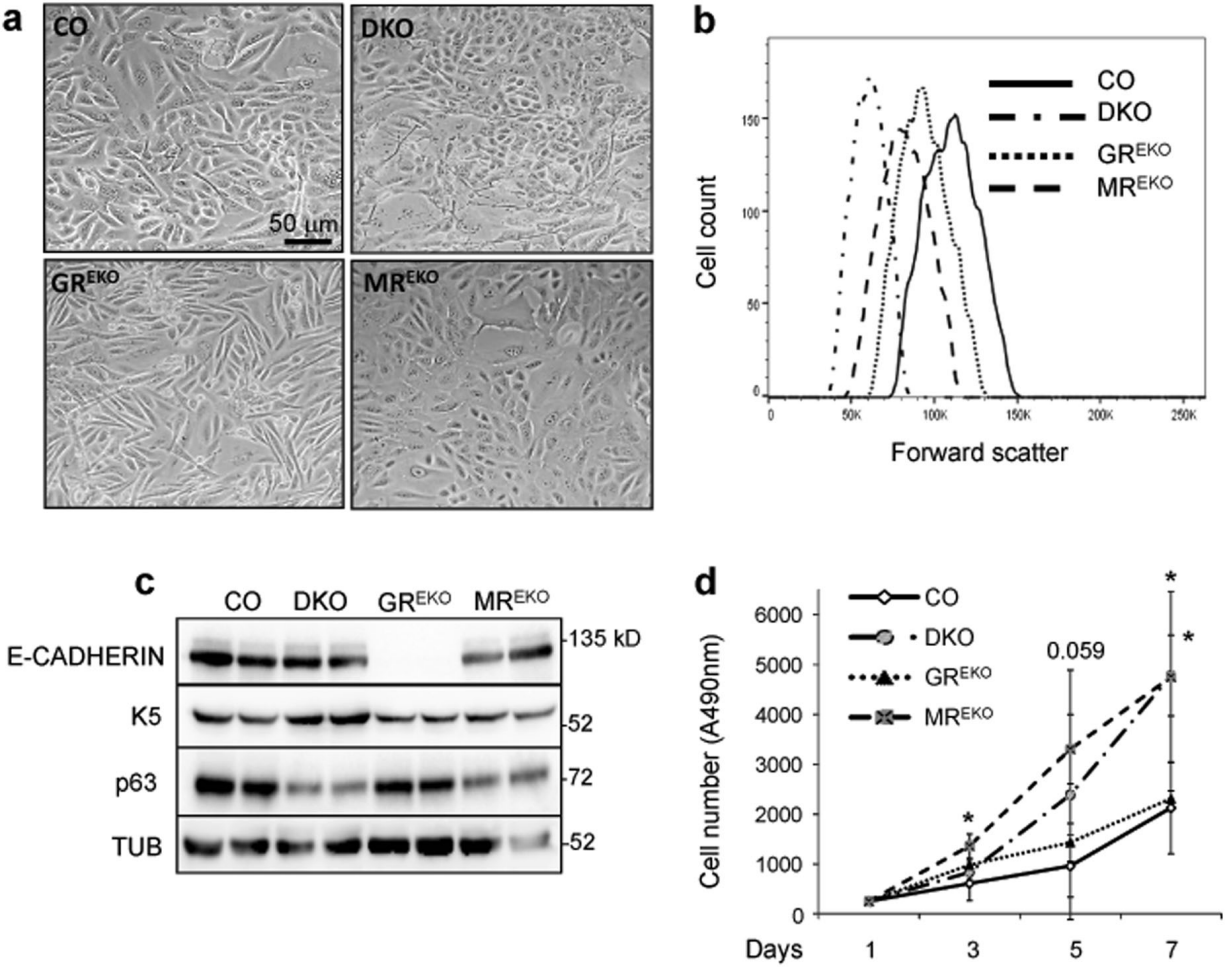
Immortalized DKO keratinocytes show a unique phenotype relative to GR^EKO^, MR^EKO^, and CO cells, with reduced cell size and altered proliferation. **a** Representative phase contrast images of CO, DKO, GR^EKO^, and MR^EKO^ keratinocytes. **b** Representative experiment for forward and side scatter evaluated by flow cytometry in CO, DKO, GR^EKO^, and MR^EKO^ cells; *n* = 3. **c** Immunoblotting of cell lysates using E-cadherin, K5, p63, and tubulin antibodies. **d**. Growth kinetics of cultured CO, DKO, GR^EKO^, and MR^EKO^ keratinocytes. Asterisks indicated statistically significant differences relative to CO: *p < 0.05; *n* > 4 biological replicates per genotype

DKO keratinocytes showed reduced cell size relative to CO or single KO, which was confirmed by flow cytometry (Fig. 4a, b). The expression of the epithelial-specific marker E-cadherin was absent only in GR^EKO^ keratinocytes consistent with its previously reported epithelial–mesenchymal transition phenotype^24^. The downregulation of E-cadherin in GR-deficient but not DKO cells suggests that keratinocyte alterations are not due to intrinsic GR deficiency but rather to a pathological role of MR in the absence of GR (Fig. 4c).

The expression of K5 was upregulated while the epithelial marker p63 was downregulated in DKO relative to the other cell lines (Fig. 4c). Overall, these data suggest that both GR and MR are required in keratinocytes for the proper expression of epithelial markers^7,8^, although their individual or combined loss does not impede the expression of the keratinocyte marker K5.

Examination of the growth rate of CO, single KO and DKO keratinocytes in culture revealed higher proliferation of DKO and MR^EKO^ cells relative to GR^EKO^ and CO with statistical significance at day 7 (Fig. 4d). These data are consistent with the known anti-proliferative role of GR and MR in epidermis^15,16,24^.

### Lack of GR and MR results in dysregulation of the MAPK/MAPK phosphatase and NF-κB pathways in keratinocytes

It is well known that MAPKs positively regulate keratinocyte proliferation and also that GC-activated GR inhibits MAPK signaling^14,25^. GCs also control the gene expression of negative MAPK regulators including the *Dusp1*/*Mkp1* and *Dusp4*/*Mkp2*^26^.

**Fig. 5:**
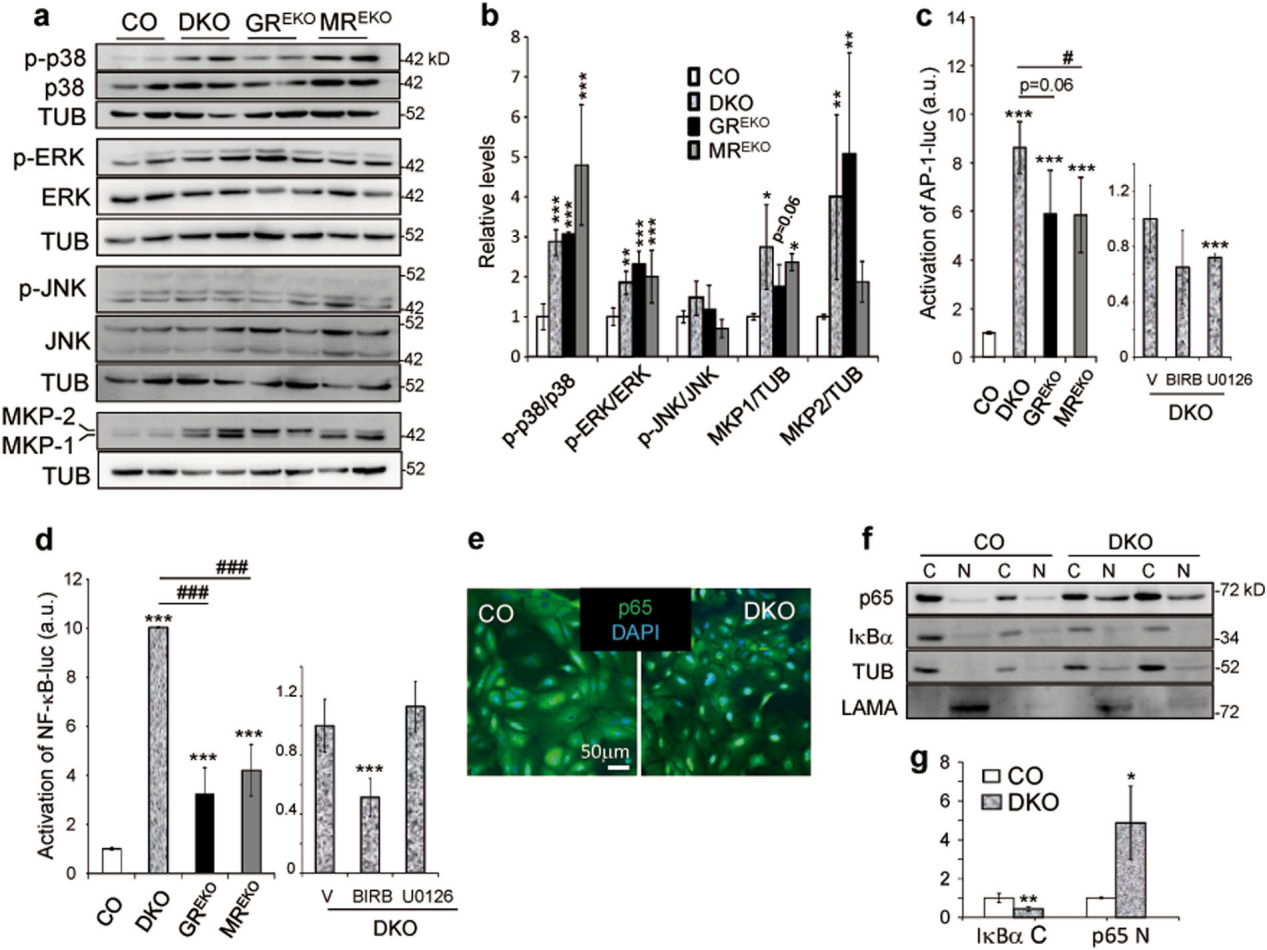
Constitutively dysregulated MAPK/MKP pathways and over activation of the AP-1 and NF-κB pathways in epidermal keratinocytes upon GR/MR loss. **a** Immunoblotting for p-p38, p38, p-ERK, ERK, p-JNK, JNK, MKP1/MKP2, and tubulin in cell extracts from CO, DKO, GR^EKO^, and MR^EKO^ cultured keratinocytes. **b** Quantitation illustrates p-p38/p38, p-ERK/ERK, p-JNK/JNK, MKP1/tubulin, and MKP2/tubulin ratios in all cell lines relative to CO. Asterisks indicate statistically significant differences relative to CO *p < 0.05, **p < 0.01, ***p < 0.001; *n* > 4 biological replicates per genotype. **c**, **d** CO, DKO, GR^EKO^, and MR^EKO^ cells were transfected with AP-1-luciferase (**c**) or NF-κB-luciferase (**d**) plasmids to assess reporter activities. DKO cells were also treated with vehicle (V), the p38 inhibitor BIRB196 (BIRB), or the ERK inhibitor U0126 as indicated. Differences relative to CO (or V) are indicated by asterisks and those between single KOs and DKO by hash signs: ^#^*p* < 0.05; ***^,###^*p* < 0.001; *n* > 4 biological replicates per genotype. **e** Immunofluorescence for p65 (green) in CO and DKO cells counterstained with DAPI (blue). **f** Immunoblotting for p65, IκBα, tubulin and laminin A (LAMA) of cytoplasmic (C) and nuclear (N) protein. **g** Quantitation relative to corresponding cell fractionation controls. Asterisks denote statistically significant differences relative to CO: *p < 0.05; **p < 0.01; *n* = 3 biological replicates per group

We checked the total and phosphorylated protein levels of MAPKs in CO, DKO, GR^EKO^, and MR^EKO^ cells (Fig. 5a). In single KO cells there were statistically significant increases of the p-p38/p38 and p-ERK/ERK ratios relative to CO indicating that both receptors are required to control these MAPKs. Also, as DKO cells showed quantitatively similar changes compared to single KO, our data suggest that GR and MR target these MAPKs through common pathways. In contrast, JNK activity was unchanged among cell lines (Fig. 5a, b).

We found that the absence of MR in MR^EKO^ or DKO cells, but not GR, correlated with statistically significant upregulation of MKP-1 (Fig. 5a, b). Conversely, in the absence of GR, MKP-2 was upregulated to a similar extent in GR^EKO^ and DKO keratinocytes while it was unchanged in MR^EKO^ cells. While MKP-1 targets preferentially p38 and JNK, MKP-2 preferably dephosphorylates ERK and JNK^15^. Previous studies in keratinocytes showed that GCs transcriptionally induced *Mkp1* while they downregulated *Mkp2*^27^. However, it has not been addressed whether this regulation is mediated by MR or GR.

Therefore, we investigated whether the changes in protein levels of these phosphatases were due to the lack of transcriptional control by GR and/or MR. Dex strongly induced *Mkp-1* in CO and MR^EKO^ cultured keratinocytes but not in GR^EKO^ or DKO, suggesting that GR is necessary and sufficient for the full induction of this gene (Fig. S7). On the other hand, Dex repressed *Mkp-2* only in CO cells, indicating that both GR and MR are required for its regulation (Fig. S7). These findings indicate that GR and MR have specific roles in modulating the expression of MKP-1/2 in epidermal keratinocytes. In addition, MKP-1/2 upregulation in DKO keratinocytes was not a direct consequence of p38 or ERK over activation since treatment with specific pharmacological antagonists did not modify phosphatase levels (Fig. S8).

We next assessed downstream AP-1 activity using luciferase reporter assays and found constitutive increases in single KO (6-fold) and DKO cells (9-fold) relative to CO (Fig. 5c), demonstrating that both receptors are required for regulation of this inflammatory signaling pathway. The increase in activity (1.5-fold) observed between DKO and single KO cells indicates cooperation between GR and MR. The pharmacological inhibition of ERK but not p38 partially ameliorated the AP-1 activity in DKO cells (Fig. 5c). The increased AP-1 activity in DKO cells even in the absence of active ERK may reflect transcriptional repression mediated by tethering of GR/MR to AP-1 or the lack of induction of other negative repressors of this pathway. Moreover, these mechanisms could account for the differences in AP-1 activity observed between single KO and DKO cells, despite having similar levels of ERK and p38 activation^28^.

Our recent work demonstrated that similar to GR, MR has anti-inflammatory roles in inflamed skin at least partially through inhibition of the NF-κB activity in keratinocytes^16^. Consistent with this, in single KO cells, NF-κB-luciferase activity was constitutively augmented by 3- to 4-fold relative to CO and, remarkably, DKO cells showed a 10-fold increase, indicating synergistic regulation by GR and MR (Fig. 5d). Inhibition of p38 but not ERK resulted in significant decrease in NF-κB activity (Fig. 5d). Cell fractionation and immunofluorescence analyses demonstrated a significant increase in nuclear p65 and a decrease in IκBα in DKO vs. CO keratinocytes, another cause for the elevated NF-κB activity in these cells (Fig. 5e-g).

### Reinsertion of GR and MR in immortalized DKO keratinocytes results in physical and functional interactions that restore the transcriptional response to GCs

**Fig. 6:**
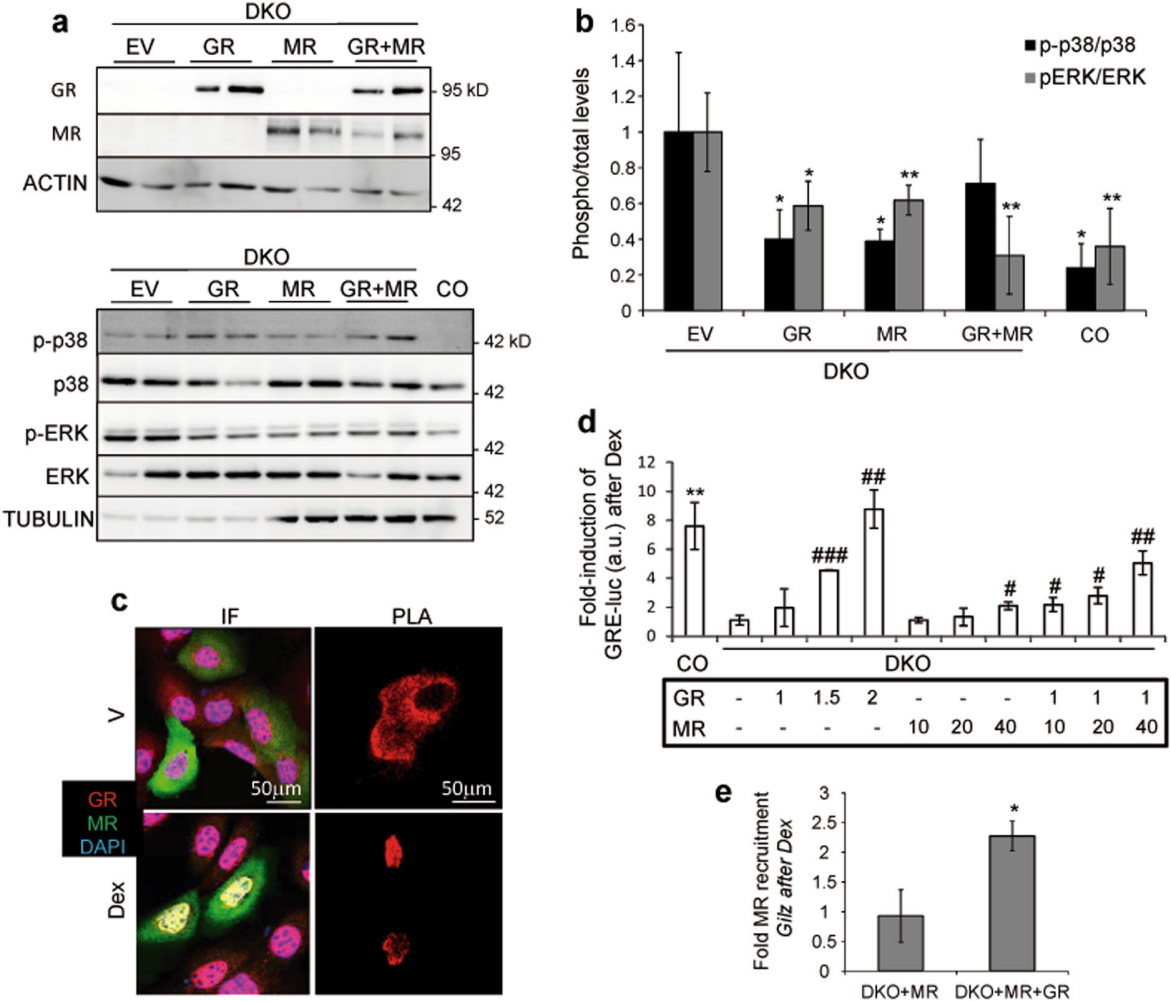
Reinsertion of GR and MR in DKO keratinocytes partially reverses elevated MAPK activity and restores the transcriptional response to GCs. **a** Immunoblotting with lysates of DKO cells transfected with empty vector (EV), GR, MR, or both receptors, demonstrating expression of GR and MR. Actin was used as a loading control. Immunoblotting for p-p38, p38, p-ERK, ERK, and tubulin in cell extracts from transfected DKO cells. CO cells were used as an internal reference. **b** Quantitation illustrates p-p38/p38, and p-ERK/ERK ratios in transfected DKO cells and CO cells. Statistically significant differences relative to EV-transfected DKO cells are indicated by asterisks: *p < 0.05; **p < 0.01; *n* > 3 biological replicates per group. **c** Immunofluorescence for HA or GFP in DKO keratinocytes transfected with HA-GR or GFP-MR and treated with vehicle (V) or Dex. Proximity ligation assays (PLA) in DKO cells co-transfected with both HA-GR and GFP-MR revealed physical interaction of the receptors in the absence and presence of hormone. **d** Luciferase assay of DKO cells transfected with indicated relative amounts of GR and MR expression vectors along with GRE-luciferase plasmid then treated with vehicle or Dex. CO cells were used as a reference. Hashes denote statistically significant differences in GR and/or MR transfected relative to EV DKO transfected cells; asterisk shows significant transactivation upon Dex treatment in CO cells: ^#^*p* < 0.05; ^##^^,^**p < 0.005; ^###^*p* < 0.001; *n* > 5 biological replicates per group. **e** Chromatin immunoprecipitation-QPCR to assess MR recruitment to GRE-containing *Gilz* regulatory sequences assays in DKO keratinocytes expressing MR or MR + GR after treatment with vehicle (V) or Dex (100 nM) for 2 h. *p < 0.05; *n* = 3

To unequivocally demonstrate that the impaired activation of the MAPK pathway is due to the loss of GC receptors, we attempted to reverse the relative increases in p38 and ERK activities by reinsertion of empty vector (EV), GR, MR, or both, into DKO cells (Fig. 6a). The expression of either receptor resulted in significant decreases in p38 and ERK activities which reached similar levels to those in CO keratinocytes (Fig. 6a, b). However, the co-transfection of both receptors did not achieve further decrease, which is consistent with the fact that the loss of either or both receptors has the same consequence for MAPK activity (Fig. 5a).

We next assessed GC-induced nuclear translocation of GR and MR by co-transfection of both receptors into DKO cells followed by Dex treatment. Immunofluorescence demonstrated nuclear co-localization of GR and MR (Fig. 6c, IF). Also, Proximity ligation assays (PLA) revealed physical interaction of GR and MR in the cytoplasm even in the absence of hormone and, upon Dex treatment this interaction was detected specifically in the nuclear compartment (Fig. 6c).

The formation of GR- and MR- homo- and hetero-dimers has been previously reported^29,30^. To decipher the relative contribution of GR and MR dimers in the transcriptional response to GCs in keratinocytes, we used DKO cells that were transfected with distinct amounts and ratios of each receptor along with a GRE-luciferase reporter plasmid. In CO, Dex induced transactivation of the GRE-luciferase reporter by ~7-fold while this response was abolished in DKO keratinocytes (Fig. 6d). The transfection of increasing amounts of GR into DKO cells restored the response to Dex in a dose-dependent manner while much higher doses of MR were required for transactivation of the GRE-luciferase reporter (Fig. 6d). However, while suboptimal doses of individually transfected GR or MR did not result in reporter transactivation after Dex treatment, their combined use achieved additive effects restoring the response to a similar extent as in CO cells (Fig. 6d). We also assessed MR recruitment to GRE-containing regulatory sequences in the classical GC-target *Gilz* by chromatin immunoprecipitation-qPCR assays and found that binding was only detected when GR was also present (Fig. 6e).

These data taken together with those showing physical interaction between receptors support the functional and cooperative role of GR and MR in mediating GC transcriptional responses in keratinocytes.

## Discussion

While the crucial role of GR in regulating epidermal morphogenesis, skin wound repair, cutaneous inflammation, and tumorigenesis has been reported^5^, the knowledge regarding MR function in skin biology is much more limited. Studies have focused on MR as responsible for some of the cutaneous undesired side-effects when GCs are in excess^16–18^. Likewise, it was reported that under physiological conditions, GR but not MR is essential for normal cardiac function and that the adverse effects in the cardiomyocyte-GR KO mice were due to GC-induced MR over activation (rather than to the loss of GR)^31,32^.

By using genetic and pharmacological approaches in mice and human skin explants, we and others demonstrated that blockade of the MR effectively improved GC-induced skin atrophy^5,16,18^. However, caution is advised for treating inflamed skin with GCs and MR inhibitors as their combined use decreased the anti-inflammatory actions of GCs in SDS-treated human skin explants^33^.

Given that GR^−/−^ and MR^−/−^ mice die perinatally^34,35^ assessing the combined actions of both receptors in vivo has been precluded. The generation of epidermal DKO mice has allowed us to unequivocally demonstrate the individual and combined roles of GR and MR in skin pathophysiology.

The combined deletion of epidermal GR and MR resulted in a severe skin phenotype at birth relative to single KOs suggesting cooperative and unique roles of GR and MR in modulating epidermal development and inflammation (Fig. 1). We also demonstrate that in response to PMA and IMQ, epidermal GR and MR have anti-inflammatory roles in adult skin by targeting the MAPK/AP-1 and NF-κB pathways and repressing the expression of inflammatory mediators. Also, the fact that neutrophil-containing microabscesses were only detected in DKO mice, during development or after inflammatory triggers, support cooperation between these receptors.

DKO were more susceptible to PMA and IMQ-induced psoriasis than CO while GR^EKO^ and MR^EKO^ mice showed an intermediate response. While the combined inactivation of epidermal GR and MR is not sufficient to cause spontaneous psoriasis it contributes to sustained inflammation (e.g., increased *Il17f* levels) and ultimately increases the susceptibility of DKOs to this disease (Fig. 3). In DKO skin, impaired GC signaling contributes to the lack of negative regulation of pro-inflammatory genes and a defective induction of anti-inflammatory mediators. These data are consistent with recent reports demonstrating that defective local GC production in skin—as this organ can synthesize and metabolize GCs—^36,37^, could contribute to the pathogenesis of psoriasis^38^.

Our data suggest that the role of distinct GR- and MR- homo- and hetero-dimers^29,30^ is relevant for regulating gene expression (Fig. 6). This raises the question of the underlying mechanisms for specificity given that both receptors can theoretically bind to identical GREs. One possible explanation is that differential interactions of GR or MR with other transcriptional regulators result in non-overlapping transcriptional activities. In cultured keratinocytes, either GR or MR were transcriptionally efficient in response to GCs, although GR showed much higher transactivation capability relative to MR. However, while suboptimal doses of GR and MR were individually unable to transactivate a GRE-containing reporter, co-transfection of both receptors resulted in functional interaction and additive transcriptional actions (Fig. 6).

In conclusion, we provide insights into the relative roles of GR and MR in keratinocytes by generating mice and cell lines lacking both receptors, which may be useful for better understanding the consequences of impaired GC signaling in the skin, and ultimately improving GC-based skin therapies.

## Materials and methods

### Antibodies and reagents

Unless otherwise specified all chemicals were from Sigma. Antibodies were from Biolegend: HA (MMS-101P), K5 (PRB-160P), K6 (PRB-169P), Loricrin (PRB-145P); Santa Cruz Biotechnology: p38α (sc-535), p63 (sc-8431), p65-NF-κB (sc-372), ERK (sc-154), GR (sc-1004), HSD11B2 (sc-20176), IκB(sc-371), Mkp1/2 (sc-1102), MR (sc-11412, ChIP), LaminA (sc-6214); Abcam: MR (ab64457); Cell Signalling Technology: p-ERK (Thr202/Tyr204; #4376), p-p38 (#9211S and #4631 for immunohistochemistry), p-JNK (#9251), JNK (#9252); ThermoFisher: GFP (A6455); Sigma: Tubulin (T6199), Actin (A2066); Roche: BrdU (11170376001) and BD Biosciences: E-cadherin (610181), Ly6G (551459). Secondary biotin-conjugated anti-rabbit, anti-mouse and anti-rat antibodies (Jackson ImmunoResearch) and secondary Alexa Fluor® anti-rabbit (555, A-31572) antibody (ThermoFisher) were used for immunostaining. Secondary peroxidase-conjugated anti-rabbit (NA934), anti-mouse (NA931) antibodies (GE Healthcare), and anti-goat (Jackson Immunoresearch) were used for immunoblotting.

### Animal experimentation

Animal experimentation was conducted according to current Spanish and European regulations and approved by our institution’s ethics committee (approval ID for project SAF2014-59474-R). Mice were maintained at 12 light/12 dark cycle, caged in groups (3–6 per cage), and had access to ad libitum food and water. Unless otherwise indicated, 8-week old mice in the telogen phase of the hair cycle were used for experiments and were treated and killed in the morning.

MR^loxP/loxP^//GR^loxP/loxP^ mice were generated by intercrossing MR^loxP/loxP^ with GR^loxP/loxP^ animals^39,40^. To generate double GR/MR epidermal knockout mice (DKO; keratin 5 (K5)-Cre//MR^loxP/loxP^//GR^loxP/loxP^), K5-Cre//MR^loxP/loxP^ (MR^EKO^)^16^ males were bred with MR^loxP/loxP^//GR^loxP/loxP^ females. K5-Cre//GR^loxP/loxP^ (GR^EKO^) mice have been previously described^15^. The colony was maintained by crossing DKO males with MR^loxP/loxP^//GR^loxP/loxP^ females. MR^loxP/loxP^//GR^loxP/loxP^ littermates were used as experimental controls (CO).

Skin phenotype of developing and newborn CO and DKO littermates was assessed macroscopically and histologically at E18.5 (16 CO, 20 DKO), P0 (5 CO, 5 DKO), P1 (12 CO, 11 DKO), and P3 (4 CO, 4 DKO). Epidermis for preparation of RNA or protein from P0/P1 mice was isolated by incubating dorsal skin with 1 mg/ml dispase II. Samples were from 7 CO, 9 DKO, 2 GR^loxP/loxP^, 4 GR^EKO^, 2 MR^loxP/loxP^, and 4 MR^EKO^ mice.

To determine keratinocyte proliferation, mice were injected with BrdU (130 μg/g of body weight) 1 h before killing. To assess GC-mediated gene expression, dorsal skin (*n* ≥ 4 per experimental group) was treated with Vehicle (Acetone) or Dex (8 μg/200 μl) 24 h prior to killing. The phorbol ester PMA was applied to both sides of mouse ears (8 μg/50 μl) 48 h prior to killing (*n* ≥ 4 per experimental group). To test the anti-inflammatory capacity of GCs, vehicle or Dex were applied (16 μg/200 μl) to dorsal skin 24 h prior to and upon treatment with PMA (8 μg/200 μl) 48 h prior to killing (*n* ≥ 4 per experimental group).

For IMQ-induced psoriasis, mice between 8–12 weeks old, (*n* ≥ 5 per experimental group) were used and Aldara® (5% IMQ, 3 M Pharmaceuticals; 62.5 mg) was applied to dorsal skin daily for 6 consecutive days. On day 7, mice were killed. Macroscopic parameters such as skin erythema and scaling were scored daily independently on a scale from 0 to 5: 0, none; 1, slight; 2, moderate; 3, marked; 4, very marked; 5, severe.

### Histochemical analysis

Tissues collected from mice were fixed in 4% PFA or 70% ethanol and paraffin-embedded for histopathological analysis as described^15^. 4 µm-thick sections were either stained with hematoxylin–eosin or used for immunofluorescence (see below) or immunohistochemistry performed following manufacturers’ indications for antibodies. After incubation with primary and secondary biotin-conjugated antibodies, immunoreactivity was revealed using the VECTASTAIN® Elite ABC-Peroxidase Staining Kit (Vector Labs) and the sections were counterstained with hematoxylin, mounted and analyzed using a Leica DM1000 microscope, Leica EC3 camera and LAS EZ software (Leica Biosystems, Wetzlar, Germany). Quantitation of BrdU-positive nuclei was performed by counting 100 hematoxylin-stained nuclei in the basal layer of the interfollicular epidermis in 6 random areas per immunostained section. Data were expressed as percentages relative to total hematoxylin-stained nuclei (*n* ≥ 4 biological replicates per experimental group). Measurements of ear edema and epidermal thickness were done using Image J software as described^17^.

### Immunoblotting

All protein extraction buffers were supplemented with Complete protease and phosphatase inhibitor cocktails (Roche). Whole cell protein extracts from cultured keratinocytes were prepared using RIPA buffer and those from mouse tissues were prepared by pulverizing frozen tissue, then subjecting it to 3 freeze thaw cycles in 20 mM HEPES pH7.9, 0.4 M NaCl, 1 mM EDTA, 1 mM EDTA, 25% glycerol, then adding *IGEPAL*® CA-630 NP-40 to a final concentration of 1%. Nuclear cytoplasmic fractionation was carried out as described^41^. Samples (25–30 μg/lane) were boiled in Laemmli buffer, separated on SDS-PAGE and transferred to nitrocellulose membranes (GE Healthcare). Membranes were blocked and incubated with specific primary antibodies then peroxidase-conjugated secondary antibodies following manufacturers’ indications. Immunoreactive signal was detected with Pierce ECL Plus Western Blotting Substrate (ThermoFisher) and the ImageQuant 4000 Biomolecular Imager (GE Healthcare, Little Chalfont, UK). Band intensities were quantitated using Image J software. All signals were normalized to loading controls (tubulin, actin or laminA).

### RNA isolation and quantitative RT-PCR

Trizol (ThermoFisher) was used to extract RNA from cells, whole skin or epidermis. In the cases of whole skin or epidermis, a polytron (PT1600E, Kinematica, Luzern, Switzerland) was used to homogenize samples. cDNA was generated using RevertAid H Minus Reverse Transcriptase (ThermoFisher) and oligo dT primers (ThermoFisher). RT-qPCR was performed in the 7500 Fast Real Time PCR System (Applied Biosystems, Carlsbad, CA) using specific oligonucleotides (0.3 μM each) and FastStart Universal SYBR Green Master ROX (Roche). *Hprt1* was used as housekeeping gene. Oligonucleotide sequences can be found in Table S1. Technical triplicates were used; and at least three biological replicates per experiment group were assessed to calculate the mean value ± SD.

### Cell culture and treatments

Mouse keratinocyte cell lines were established following published protocols^42,43^. Briefly, keratinocytes were isolated from 8-wk-old female mouse dorsal skin and cultured on mitomycin C treated J2-3T3 feeders in type I collagen-coated flasks in DMEM-Ham’s F12 (3:1) medium (ThermoFisher) supplemented with 1.8 × 10−4 mol/l adenine, 0.35 mM calcium, 7.5% FBS Gold (Biowest), 100 U/ml penicillin/100 μg/ml streptomycin (Biowest), 2 mM glutamine (Biowest), 0.25 μg/ml amphotericin B (Biowest), 5 μg/ml insulin, 10^−10^M cholera toxin, and 10 ng/ml EGF (Peprotech). Following approximately 8 passages, spontaneously immortalized lines arose, which were maintained using the above conditions.

To evaluate response to GCs, cells were grown overnight in medium containing charcoal-stripped serum to deplete steroids, and then treated with vehicle (EtOH) or 100 nM Dex for indicated time periods. In specified experiments, cells were treated with 100 nM PMA, 10 μM BIRB196, or 5 μM U0126 (Merck Millipore).

### Plasmids, transfections, and luciferase assays

Two mouse GRalpha constructs were generated, one untagged (pcDNA3-GR) and the other with an N-terminal HA tag (pcDNA3-HAGR). The cDNA encoding GR from pSV2Wrec^44^ was amplified by PCR and then subcloned into the *Bam*H1-*Xho*I sites of pcDNA3 (Invitrogen). Constructs were sequence verified prior to use (Table S1).

Keratinocytes at 70–90% confluence were transfected with combinations of the following plasmids: pcDNA4 (ThermoFisher), pcDNA4-MR^45^, pcDNA3-GR, pGRE2EIB-Luciferase^46^, pGL3-NF-KB-5×-Luciferase^47^, −73/+63 Collagenase-Luciferase^48^, and the internal control pRL-SV40 Renilla (Promega) using Lipofectamine 2000 Reagent (ThermoFisher). Luciferase activity was measured using the Dual-Luciferase® Reporter Assay System (Promega) and a Wallac 1420 Victor2 Microplate Reader (Perkin Elmer, Waltham, Massachusetts). Firefly-luciferase levels were normalized to those of Renilla luciferase.

### Proliferation assays

Overall, 250 keratinocytes were plated per well (*n* = 4 per genotype and time point) in 96-well plates on day 0, 3, 5, and 7, 20 µl per well of CellTiter 96® AQueous One Solution Cell Proliferation Assay Reagent (Promega) was added and incubated for 3 h at 37 °C. Absorbance was measured at 490 nm using a Wallac Victor2 1420 multilabel counter (Perkin Elmer).

### Flow cytometry

Following trypsinization, resuspension in serum-containing media, and washing with PBS, cultured keratinocytes were resuspended in DPBS + 2% BSA. Forward and side scatter were evaluated using a BD FACS Canto Flow Cytometer (Franklin Lakes, New Jersey) and data were analyzed using FlowJo Software (BD Biosciences).

### Immunofluorescence and proximity ligation assay (PLA)

Keratinocytes were cultured on collagen I-coated coverslips, fixed with 4% PFA and permeabilized with 0.2% Triton X100 in PBS prior to blocking with 5% donkey serum in PBS and overnight incubation with primary antibodies at 4 °C. The following day, samples were washed with PBS, and incubated 1 h with secondary antibodies and DAPI (ThermoFisher). The same protocol, omitting the permeabilization step, was used for dewaxed sections of paraffin-embedded EtOH-fixed mouse skin. Samples were mounted using Mowiol (Calbiochem) and DABCO and then analyzed using a Leica DM RXA2 fluorescent microscope.

Proximity ligation assays (PLA) were performed as described with PLA Duolink® In Situ Red Starter Kit Mouse/Rabbit (Sigma)^49^. Briefly, for detection of GR/MR interactions, GFP-MR^44^ and HA-GR constructs were transfected into DKO cells at a 1:1 ratio, then cells were treated with Dex 100 nM for 16 h. Protein interaction was detected using anti-GFP and anti-HA primary antibodies diluted in Antibody Diluent solution overnight at 4 °C. As negative controls, cells transfected with either GFP-MR or HA-GR were used.

### Chromatin immunoprecpitation

MR chromatin immunoprecipitations were performed as described using DKO keratinocytes transfected with 1:1 ratios of pcDNA3-empty vector and pcDNA4-MR or pcDNA3-GR and pcDNA4-MR^50^. QPCR was performed to determine the relative amplification of sequences corresponding to a *Gilz* enhancer in Dex- vs. vehicle-treated chromatin immunoprecipitations, which were normalized to the amplification values of respective inputs (primers; Supplementary Table S1).

### Statistical analysis

Experimental data were analyzed using IBM SPSS Statistics software. In all graphs, mean values ± SD are shown. When statistical analysis was performed with relative values, data were first subjected to logarithmic transformation. Prior to parametric testing, the Levene’s test was used to determine whether samples within groups had equal variance. For comparisons between two experimental groups, we used the Student’s unpaired two-tailed *t*-test. For comparisons among more than two experimental groups, we used the one-way ANOVA, which if statistically significant was followed by a post hoc Tukey multiple comparison test. *P* values <0.05 were considered statistically significant.

## Electronic supplementary material

Supplemental Material
