# Supplementary material for: Epidermal glucocorticoid and mineralocorticoid receptors act cooperatively to regulate epidermal development and counteract skin inflammation
Source: Cell Death Dis. 2018 May 22;9(6):588. doi: 10.1038/s41419-018-0673-z (PMC5964110; doi:10.1038/s41419-018-0673-z)
Supplement: Supplementary file 1 — Supplemental Material [file 41419_2018_673_MOESM1_ESM.pdf]

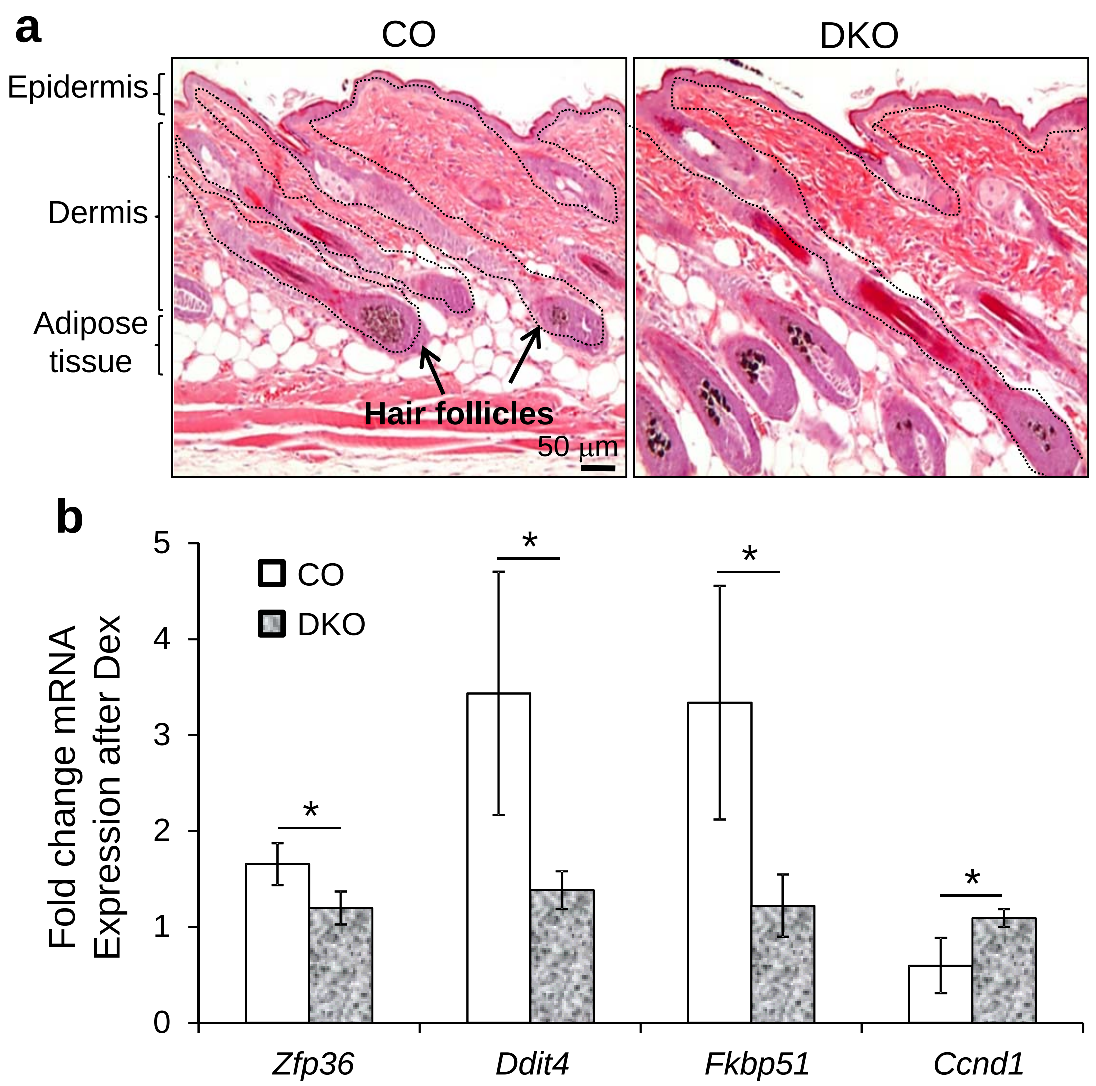

Fig. S1. DKO adult mice feature relatively normal skin architecture but lack response to topical GC treatment.

(a) Representative images of H&E-stained sections from CO and DKO adult dorsal skin. (b) Gene expression was quantitated by RT-QPCR using dorsal skin from CO and DKO littermates treated with vehicle or dexamethasone (Dex) for 24h. Asterisks indicate statistically significant differences between genotypes in the fold change of gene expression upon Dex treatment: *, $p < 0.05$; $n \geq 3$ mice per genotype and treatment.

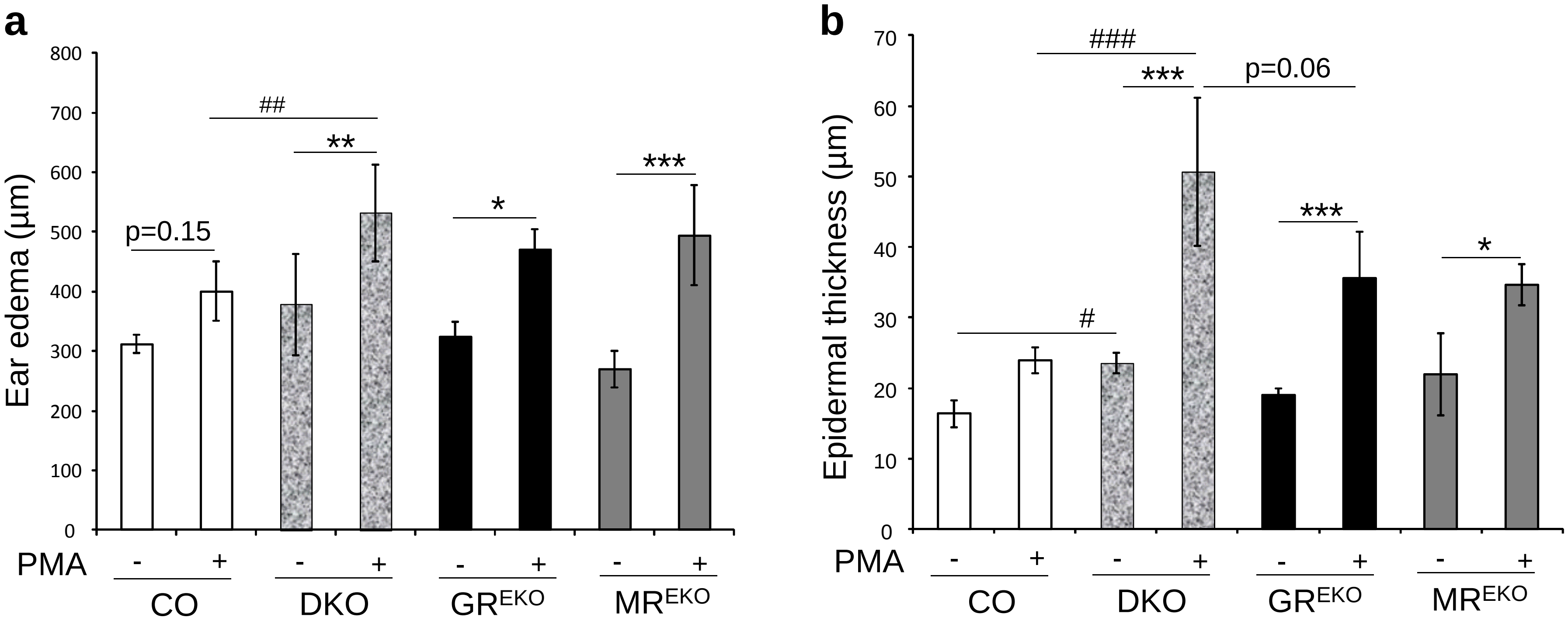

Fig. S2. PMA-induced ear edema and epidermal thickness in CO, DKO, GR^{EKO}, and MR^{EKO} mice. Quantitation of ear edema and epidermal thickness after 48h topical treatment of CO, DKO, GR^{EKO}, or MR^{EKO} mice with vehicle (-) or PMA (16 mg/ear). Asterisks denote statistically differences between treatment groups, and hash sign statistically significant differences between genotypes: *, # $p<0.05$, **, ## $p<0.01$, ***, ### $p <0.001$. $n \geq 4$ per genotype and treatment.

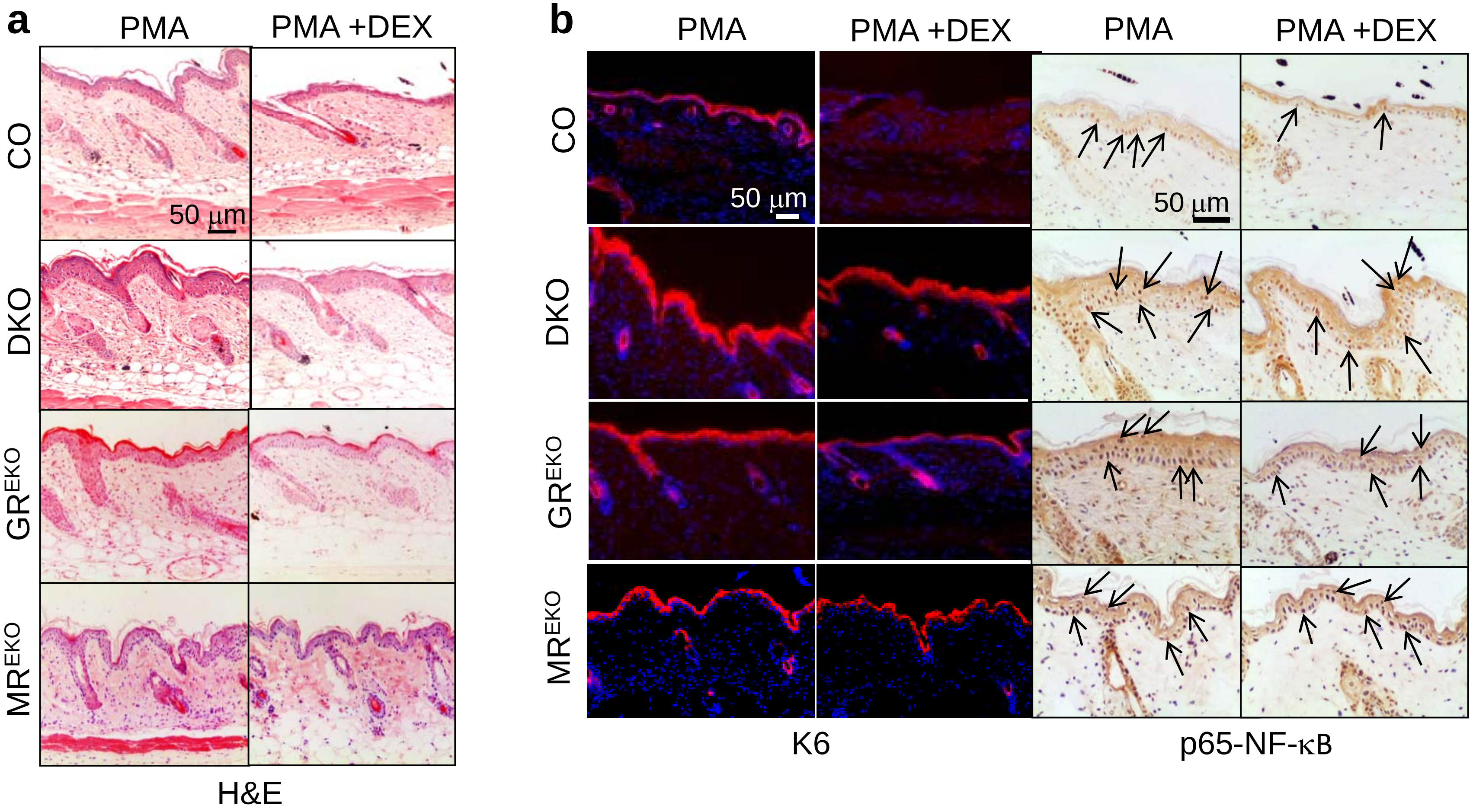

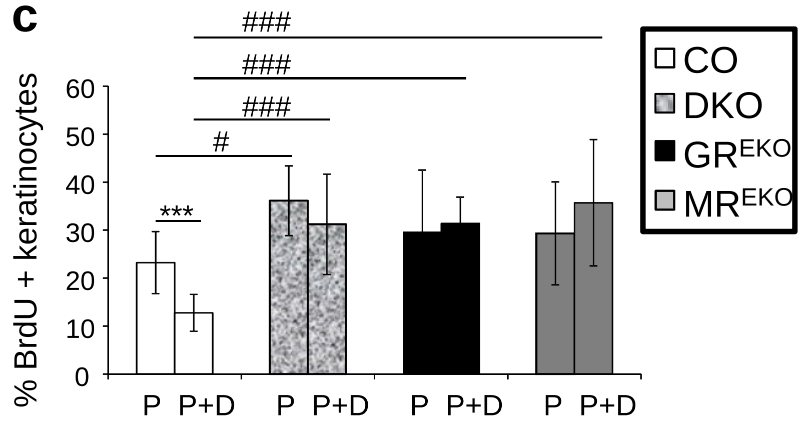

<u>Fig. S3. PMA response is only partially ameliorated by Dex in DKO and single KO relative to CO mice.</u>

(a) H&E-stained and (b) immunostaining of skin sections from PMA or PMA+Dex-treated mice to assess K6 (red; DAPI, blue) and p65-NF-κB expression. (c) Quantitation of epidermal keratinocyte proliferation by BrdU labeling following PMA (P) or PMA+Dex (P+D) treatment. Statistically significant differences between treatments within one genotype are denoted by asterisks, and comparison between genotypes within the same treatment groups are indicated by hashes: #, $p < 0.05$; ***, ###, $p < 0.001$; $n \geq 4$ mice per experimental group.

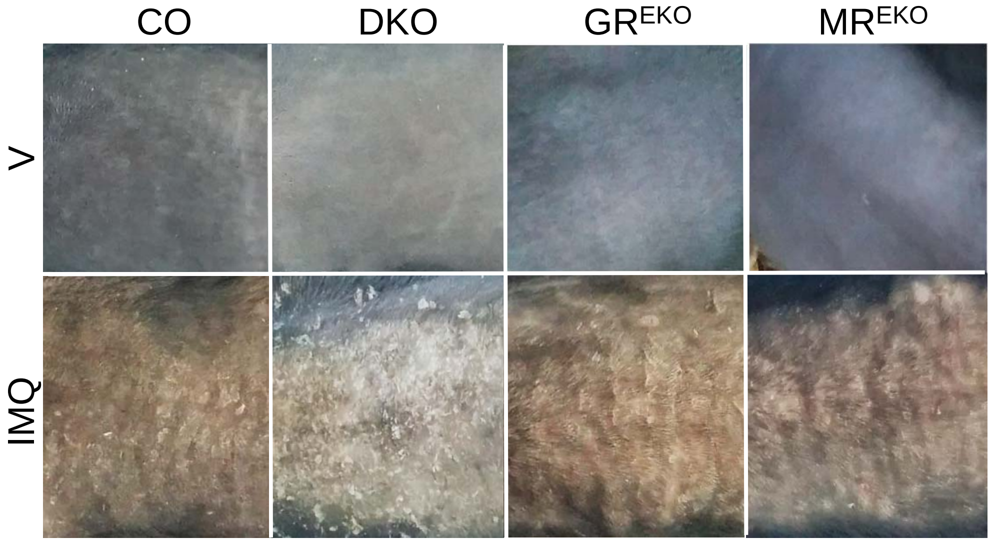

Fig. S4. Increased scaling in IMQ-treated DKO relative to single KO and CO mice. Representative images illustrate scaling in CO, DKO, GR^{EKO}, or MR^{EKO} mice at experimental end-point. V, vaseline; IMQ, imiquimod treatment.

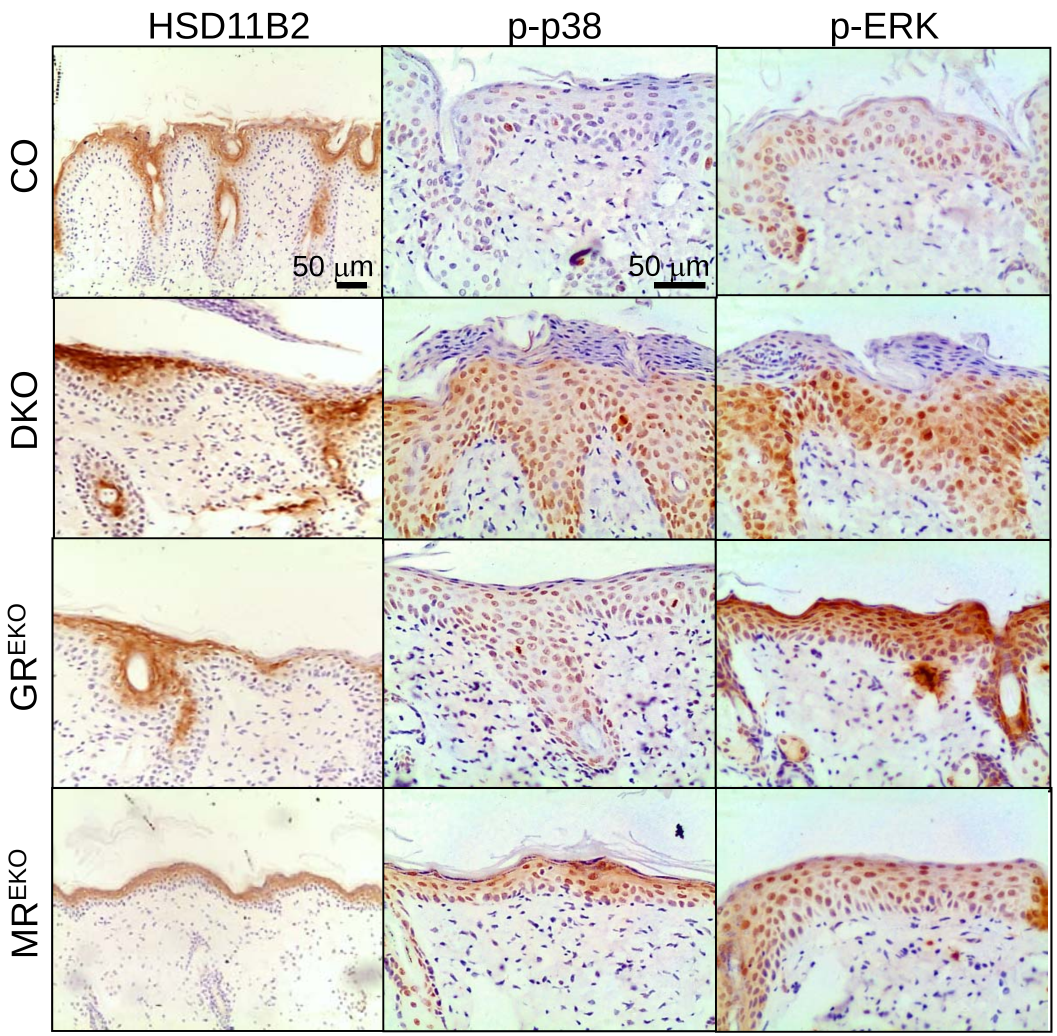

Fig. S5. Altered expression of HSD11B2, p-p38, and p-ERK in DKO and single KO relative to CO mice after IMQ treatment.

Representative images illustrate immunostaining for HSD11B2, p-p38, and p-ERK in CO, DKO, GR^{EKO}, or MR^{EKO} mice at experimental end-point.

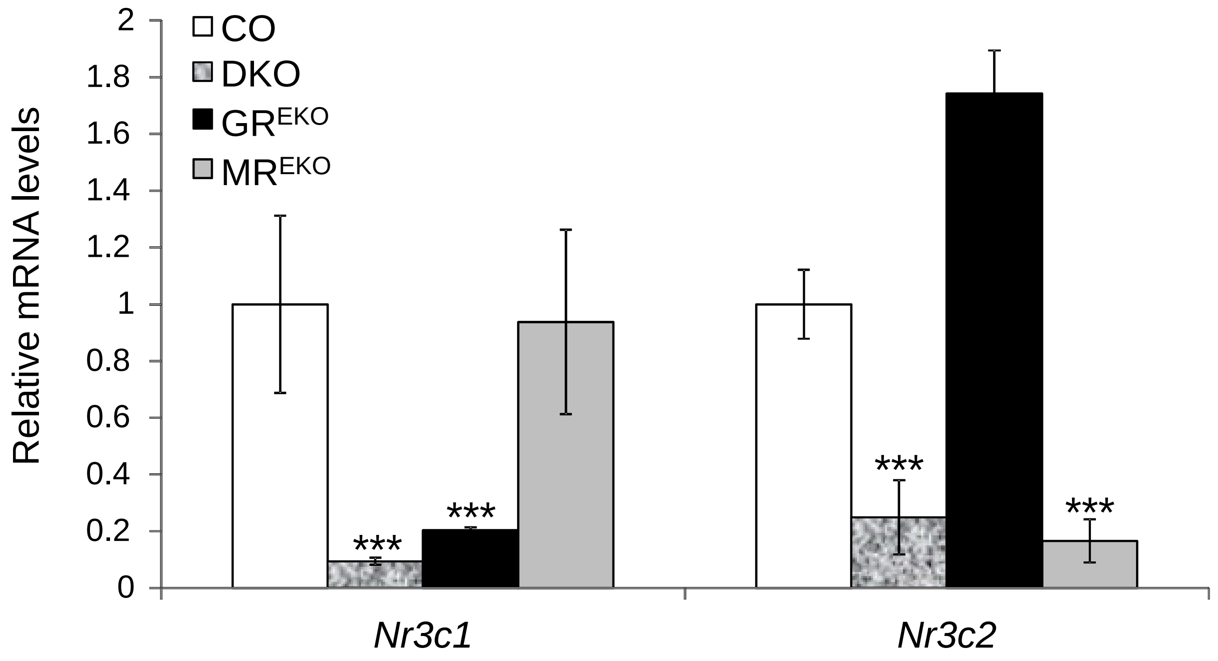

Fig. S6. Relative *Nr3c1* and *Nr3c2* mRNA levels in CO, DKO, GR^{EKO}, and MR^{EKO} cell lines.

The relative mRNA levels of *Nr3c1* and *Nr3c2* were quantitated by RT-QPCR in the indicated cell lines. Asterisks indicate statistically significant differences relative to control: ***, $p < 0.001$; $n \geq 4$ biological replicates per genotype.

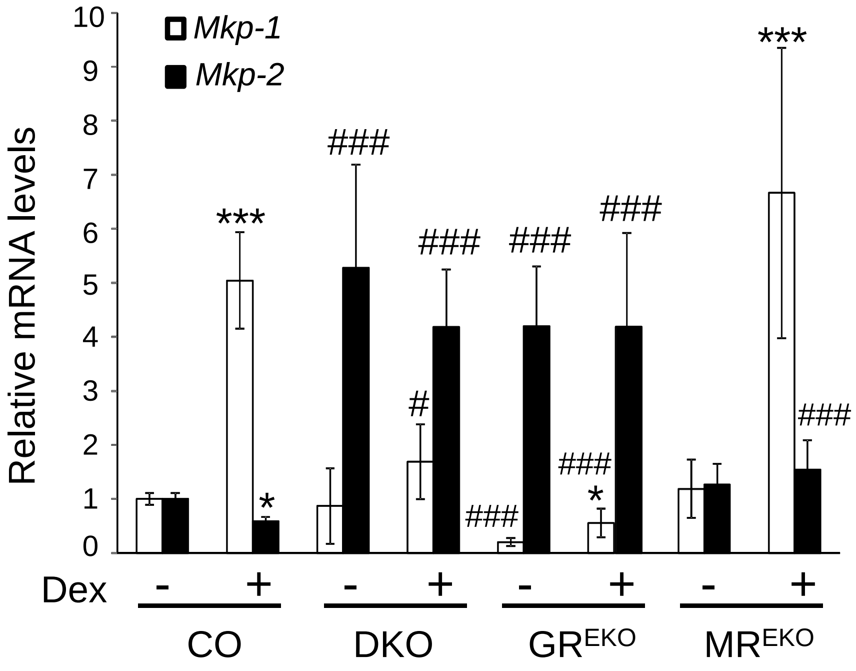

Fig. S7. *Dusp1/Dusp4* mRNA expression in cells treated with vehicle or Dex.
The relative mRNA levels of *Dusp1/Mkp1* and *Dusp4/Mkp2* were quantitated by RT-QPCR in CO, DKO, GR^{EKO}, and MR^{EKO} keratinocytes after culture in charcoal-treated serum, then treatment with vehicle or Dex (100 nM, 3h). Statistically significant differences within each genotype in response to ligand are indicated by asterisks and those relative to CO (the same treatment group) with hash signs: *, #, $p < 0.05$; ***, ###, $p < 0.001$; $n \geq 4$ biological replicates per genotype.

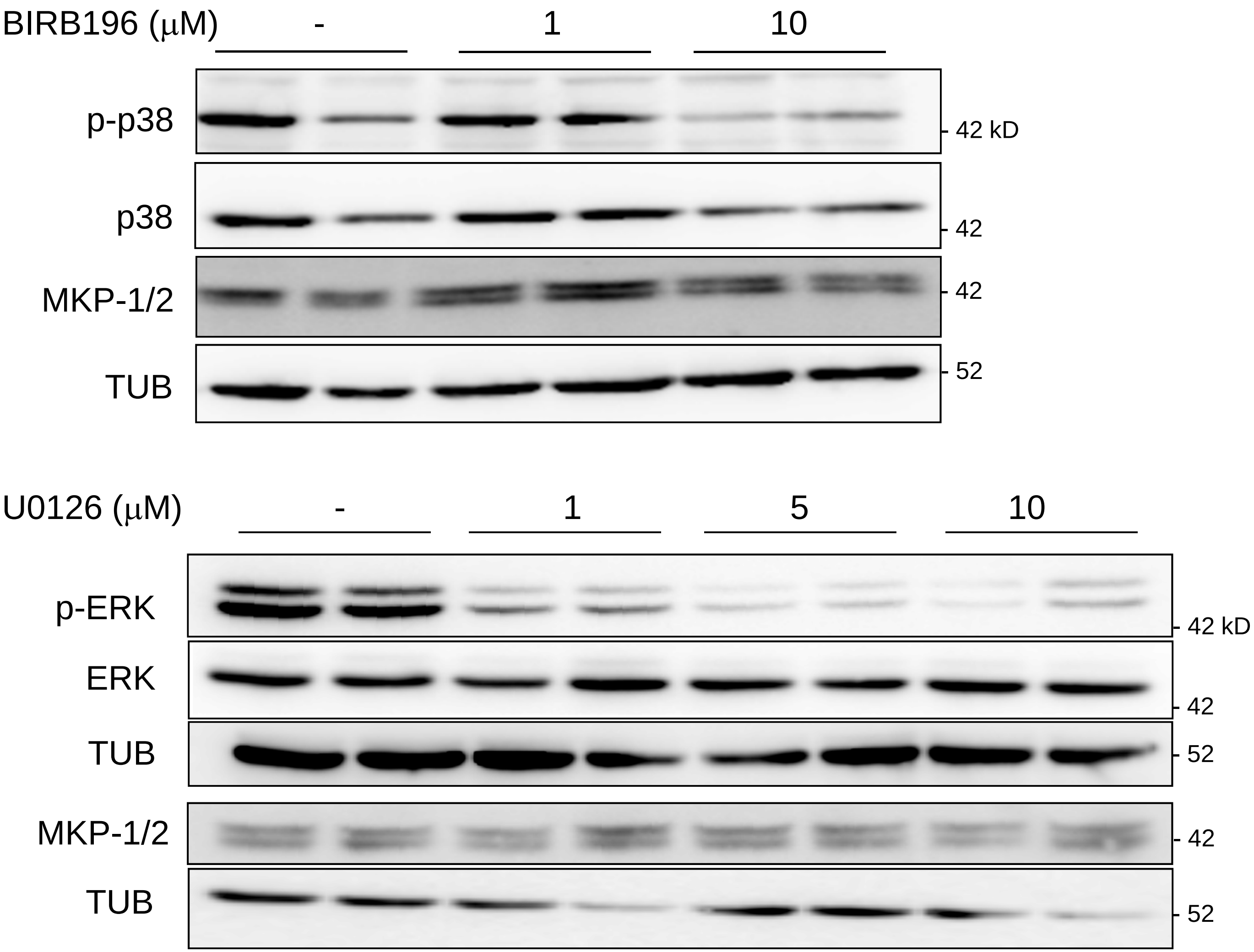

Fig. S8. MKP1/2 up-regulation is not due to ERK or p38 overactivation in DKO keratinocytes.

Immunoblotting for p-p38, p38, p-ERK, ERK, MKP1/MKP2, and tubulin in cell extracts from DKO keratinocytes in the absence (-) or presence of the indicated concentrations of inhibitors BIRB196 or U0126.

Table S1

Primers used for RT-QPCR

Gene	Forward	Reverse	Product size
Ccnd1	CATCAAGTGTGACCCGGACTG	CCTCCTCCTCAGTGGCCTTG	116
Ddit4	GGGCCGGAGGAAGACTCCTCATA	CTGTATGCCAGGCGCAGGAGTTC	218
Dusp1	CTCCACTCAAGTCTTCTTTCTCC	TAGGCACTGCCCAGGTAC	219
Dusp4	GTCGAAGACAACCACAAGGC	CGCCCTCGACAGTCCTTTAC	86
Fkbp51	CTTGGACCACGCTATGGTTT	AACGACTCTGAGGCTTTGGA	290
Hprt1	TCAGTCAACGGGGGACATAAA	GGGGCTGTACTGCTTAACCAG	142
Hsd11b1	GGAGCCGCACTTATCTGAA	GACCTGGCAGTCAATACCA	253
Hsd11b2	CTGCAGATGGATCTGACCAA	GTCAGCTCAAGTGCACCAAA	197
Il17f	CCCAGGGCTGTTCTAATTCCTT	GACACAGGTGCAGCCACCTTT	65
Il6	GATGCTACCAAACTGGATATAATC	GGTCCTTAGCCACTCCTTCTGTG	269
Krt77	GAGCAAAGATGAGGCTGAGG	CCTCCGCATCAGAAATCAAT	200
Lcn2	GGCCCTGAGTGTCATGTGTC	TTCTGATCCAGTAGCGACAGC	308
Mmp3	CCAGGTGTTGACTCAAGGGTGGA	ACACAGGATGCCTTCCTTGGATCTC	172
Nr3c1	TGCTATGCTTTGCTCCTGATCTG	TGTCAGTTGATAAAACCGCTGCC	299
Nr3c2	GTGGACAGTCCTTTCACTACCG	TGACACCCAGAAGCCTCATCTC	286
S100a8	GGAGTTCCTTGCGATGGTGAT	TCTGCTACTCCTTGTGGCTGTCT	68
Slpi	TGGCACCCTGGACTGTGGAAGG	CCCGTCCCTCTGGCAGACAT	278
Sprr2d	TGGTACTCAAGGCCGAGA	TTTGTCCTGATGACTGCTGAAGAC	336
Tslp1	CCAGGCTACCCTGAAACTGA	TCTGGAGATTGCATGAAGGA	125
Zbtb16	CCCAGTTCTCAAAGGAGGATG	TTCCCACACAGCAGACAGAAG	88
Zfp36	CCACCTCCTCTCGATACAAGA	GCTTGGCGAAGTTCACCCA	112

Primers used in PCR subcloning into pcDNA3

cDNA	Forward	Reverse	Product size	Restriction sites
GR	GCGGGATCCGCCACCATGGACTCCAAAGAAT	GCGCTCGAGTCATTTCTGATGAAACAGAAGC	2376	*BamHI* 5', *XhoI* 3'
HA-GR	GCGGGATCCGCCACCATGTACCCCTACGAC[illegible]	GCGCTCGAGTCATTTCTGATGAAACAGAAGC	2439	*BamHI* 5', *XhoI* 3'

GR sequencing primers

Binds		
nt 555	Sequence	Strand
nt 1153	GCAAAATAGAAAAAGCCAGCC	forward
nt 1900	CAAGGGTCTGGAGAGGACAA	forward
nt 1846	AACCTGCTATGCTTTGCTCC	forward
nt 1255	TCCATGAGTACTGTAGAAGGGTCA	reverse

Primers used in chromatin immunoprecipitation QPCR

Gilz	Forward	Reverse	Product size
	GGAGGGAATGCAACTGGGAG	CCCCTCCCTTGAATGCTGAA	91
